# Simultaneous Endovascular Repair Is Not Associated With Increased Risk for Thoracic and Abdominal Aortic Pathologies: Early and Midterm Outcomes

**DOI:** 10.3389/fcvm.2022.883708

**Published:** 2022-05-27

**Authors:** Weichang Zhang, Lei Zhang, Xin Li, Ming Li, Jian Qiu, Mo Wang, Chang Shu

**Affiliations:** ^1^Department of Vascular Surgery, The Second Xiangya Hospital, Central South University, Changsha, China; ^2^Institute of Vascular Diseases, Central South University, Changsha, China; ^3^Department of Cardiovascular Surgery, Chinese Academy of Medical Sciences and Peking Union Medical College Fuwai Hospital, Beijing, China

**Keywords:** thoracic endovascular aortic repair (TEVAR), EVAR, spinal cord injury (SCI), abdominal aortic aneurysm (AAA), aortic pathologies, simultaneous

## Abstract

Coexisting multilevel aortic pathologies were caused by atherosclerosis and hypertension and presented in a small subgroup of patients. Endovascular repair is a safe and effective treatment for a variety of aortic pathologies. However, fewer small series and cases were reported using simultaneous thoracic endovascular repair (TEVAR) and endovascular aneurysm repair (EVAR) for both aortic segments. To determine the outcomes of simultaneous and separately TEVAR and EVAR treating for multilevel aortic pathologies. Between 2010 and 2020, 31 patients and 22 patients were treated by one-staged and two-staged repair, respectively at a single center. All patients had the concomitant thoracic and abdominal aortic disease (aortic dissection, aneurysms, and penetrating aortic ulcers). Compared with the patients with two-staged aortic repair, the one-staged repair patients were older (mean age, 68 vs. 57 years; *P* < 0.001) and had a larger preoperative maximal aortic diameter (67.03 ± 10.65 vs. 57.45 ± 10.36 mm; *p* = 0.002). The intraoperative and postoperative outcomes show that the procedure times and length of hospital stay (LOS) were longer in the two-staged group. There is no significant difference in postoperative complications between the two groups. In the follow up, the freedom from re–intervention and the mean survival rate for the one-staged group were 100 vs. 100%, 92.4 vs. 95%, and 88 vs. 88% at one, two, and 5 years, respectively, whereas the mean survival rate for the two-staged group was 86.4 vs. 90.5%, 87 vs. 90.5%, and 76 vs. 84% at one, two, and 5 years, respectively, all with no statistical difference. Combined TEVAR and EVAR can be performed successfully with minimal morbidity and mortality. The one-staged repair was not associated with the increased risk for multilevel aortic pathologies treatment.

## Introduction

Coexisting multilevel aortic pathologies are present in a small subgroup of patients, with the most frequently encountered combination being descending thoracic aortic pathologies and infrarenal abdominal aneurysms (1). Endovascular aortic repair has been shown to be a safe and effective treatment modality for a variety of aortic pathologies, with improved perioperative morbidity and mortality and similar long–term results compared with open repair (2, 3). However, there is currently no consensus about whether a combined or separated treament should be adopted in the case of multilevel aortic pathologies.

Previous studies have shown that one-staged intervention has been associated with higher complications such as spinal cord ischemia (SCI) (4). However, these findings are largely based on studies of a combination of thoracic stent grafts and open abdominal approaches (5–7). Fewer small series and case reports have evaluated concomitant endovascular repair of both aortic segments (1, 8–10). Moreover, compared to the two-stage intervention, the one-staged operation can eliminate the requirement for a second surgical intervention and the rupture risk of the residual aortic lesion while waiting for the second repair. Here, we report our experience in the simultaneous and separate treatment of endovascular stent grafts for combined thoracic and abdominal aortic disease.

## Methods

### Study Population and Design

Aortic endovascular repair cases were identified in the archives of medical records of our hospital, including thoracic endovascular aortic repair (TEVAR) and abdominal aortic endovascular repair from 2010 to 2020. Multilevel aortic diseases were included in this study, including aortic dissection (AD), penetrating aortic ulcer (PAU), thoracic aortic aneurysm (TAA), and abdominal aortic aneurysm (AAA). The exclusion criteria were isolated TEVAR or endovascular aneurysm repair (EVAR), age < 18 years, and endovascular conversion to open surgery. Thirty-one were simultaneously treated with aortic stent grafts, while 22 patients were treated separately. The mean age in one-staged and two-staged groups were 68 ± 9 and 56 ± 9 years, respectively. The demographic and cardiovascular risk factors and pre-operative comorbidities are summarized in Table 1. The entire aorta was obtained by CT angiography (CTA) to determine the appropriateness of intravascular intervention and to determine the size of the stent graft prior to surgery. The aortic lesions included AD (*n* = 5), PAU (*n* = 21), thoracic aortic aneurysm (TAA; *n* = 1), and AAA (*n* = 22). Follow-up data on outcomes of operation were collected. Mortality was assessed at in–hospital stays and postoperative 30 days.

**Table 1:** Demographics and preoperative characteristic.

**Variable**	**TEVAR simultaneous with EVAR** **(*n* = 31)**	**TEVAR separately with EVAR (*n* = 22)**	***P*-value**
Demographic and risk factors			
Age, years	68 ± 9	57 ± 9	<0.0001
Gender			
Male	27 (87.10)	17 (77.27)	0.833
Female	4 (12.90)	5 (22.73)	-
BMI, kg/m^2^	24.00 ± 3.04	23.00 ± 2.73	0.0563
Smoking history			0.645
Never smoker	2 (6.45)	3 (14.29)	-
Prior/current smoker	29 (93.55)	19 (86.36)	-
Pathology			
Abdominal aortic aneurysm	16 (51.61)	6 (28.57)	0.099
Aortic dissection	2 (6.45)	3 (14.29)	0.645
Aortic penetrating ulcers	18 (58.06)	4 (13.64)	0.002
TAAA	-	1 (4.76)	0.404
Preoperative maximal aorticdiameter, mm	67.03 ± 10.65	57.45 ± 10.36	0.0020
COPD	10 (32.26)	8 (38.10)	0.664
CKD (creatinine ≥ 1.8 mg/dL)	6 (19.35)	2 (14.29)	0.567
Dialysis	3 (9.68)	2 (9.52)	1.000
Diabetes	16 (51.61)	8 (38.10)	0.337
Hypertension	29 (93.5)	19 (86.4)	1.000
CHF	2 (6.45)	1 (4.76)	1.000
CAD	10 (32.26)	8 (38.10)	0.664
CVD	6 (19.35)	4 (19.05)	1.000

*TEVAR, Thoracic endovascular aortic repair; EVAR, Endovascular aortic repair; BMI, body mass index; CAD, coronary artery disease; CHF, congestive heart failure; CKD, chronic kidney disease; COPD, chronic obstructive pulmonary disease; CSF, cerebrospinal fluid; CVD, cerebrovascular disease; EVAR, endovascular aortic repair; LSCA, left subclavian artery; TEVAR, thoracic endovascular aortic repair. Data presented as number (%) for categorical variables and mean ± standard deviation or median for continuous variables*.

### Outcomes and Definitions

Technical success, SCI, in-hospital, and 30-day mortality were assessed as early outcomes. Survival, grafts instability, and freedom from re-intervention (FFR) were evaluated during the follow-up. The technical success was defined as endograft being deployed correctly at the planned position without iliac leg stenosis/occlusions, type I and III endoleaks, open surgical conversion, and 24 h mortality. The primary outcome was the development of postoperative SCI, which was defined according to the Society of Vascular Surgery's reporting standards (11).

The secondary outcomes were the postoperative hospital LOS, postoperative in-hospital complications, including cardiac complications, stroke, bowel ischemia, renal failure or ischemia, and perioperative mortality. The LOS, operative time, and iodized contrast agent dosage in the two-stage groups were the accumulative amounts of two operations (2). Cardiac complications included congestive heart failure, new–onset arrhythmia, or postoperative myocardial infarction. Bowel and renal ischemia were defined as end–organ malperfusion requiring either medical treatment or surgical revascularization. Worsening postoperative renal function was defined as a reduction of glomerular filtration rate of > 30% of the baseline value.

#### Follow-Up

The follow-up surveillance program was done with physical examination and CTA at 2 weeks, 3 months, 6 months, and 12 months after the operation, and annually thereafter to evaluate the position of aortic grafts and endoleaks. Survival was assessed by outpatient clinic visits or telephone interviews (12).

#### Statistical Analysis

The patients were stratified into two groups: one-staged repair and two-staged repair. Data are present as absolute numbers and percent prevalence. Categorical variables were compared using the χ^2^ test or Fischer's exact test, where appropriate. Continuous variables were reported as mean ± *SD* and compared using the Mann–Whitney U test. The categorical variables were reported as frequencies and proportions. Survival, FFR, and chimney patency were estimated by the Kaplan–Meier analysis. All the statistical tests were two-sided, and *p*-values of < 0.05 were predetermined to indicate statistical significance. Statistical analysis was performed by SPSS 23.0 for Windows (IBM, Armonk, NY, USA).

This study was approved by the Institutional Review Board at the Second Xiangya Hospital of Central South University.

## Results

Demographic, cardiovascular risk factors, and pre-operative comorbidities are summarized in Table 1. The mean diameter of the aortic lesion in one-staged and two-staged were 67.03 ± 10.65 and 57.33 ± 10.6, respectively. Twenty cases of LSCA were covered during the endovascular repair, of them,15 cases were preserved using a chimney or fenestrate techniques, and the other 5 cases were without revascularizaiton.

Stent grafts were deployed with technical success in all patients. For one-staged repair patients, TEVAR was routinely performed prior to EVAR. For two-staged repair patients, the priority of TEVAR or EVAR was depending on what is the symptom of the aortic lesion coming first. Compared with the patients with two-staged aortic repair, the one-staged repair patients were older (mean age, 68 vs. 57 years; *P* < 0.001). There is no difference in the prevalence of CKD and cardiovascular and pulmonary disease risk factors, including coronary artery disease, hypertension, chronic obstructive pulmonary disease, and positive smoking history. Compared to the two-staged repair group, the one-staged group also had a larger preoperative maximal aortic diameter (67.03 ± 10.65 vs. 57.45 ± 10.36 mm; *p* = 0.002).

### Procedure and Early Outcomes

Intra- peri-, and post-operative information are summarized in Table 2. All procedures were performed under general anesthesia through a surgical incision or percutaneously at the femoral level, if appropriate. A total of 31 cases (67.6%) were treated by one-staged repair, other 22 cases were treated by two-staged repair. The mean procedure times were 139.5 ± 24.08 and 172.04 ± 28.04 min, respectively. The mean volume of iodinated contrast medium was 96.45 ± 19.24 and 92.72 ± 14.2 ml, respectively. The patients stay in the Intensive Care Unit (ICU) and the mean hospital LOS in the one- and two-staged groups were 8.9 ± 2.97 vs. 15.81 ± 2.63 (*p* < 0.0001) and.9 ± 2.53 vs. 1.36 ± 4.1 days, respectively. Technical success was achieved in 52 cases (100%). There were two deaths, which occurred 24 h postoperatively in each group with sudden cardiac arrest. At the completion of angiography, the chimney patency was 100%. SCI occurred in two (6.5%) cases, all of them suffered transient paraparesis. Cardiac and pulmonary complications were reported in 9 and 6 cases, respectively. Cardiac complications were congestive heart failure (*n* = 1 vs. *n* = 1), new–onset arrhythmia (*n* = 3 vs. *n* = 2), and postoperative myocardial infarction (*n* = 2 vs. *n* = 2). Pulmonary complications were respiratory failure (*n* = 1 vs. *n* = 0), and pleural effusion (*n* = 0 vs. *n* = 1), and pneumonia (*n* = 2 vs. *n* = 1). Three patients suffered worsened postoperative renal function and required postoperative hemodialysis. There were two cases of bowel ischemia since the aortic dissection was affecting the superior mesenteric artery and the blood supply was from the false lumen, but the symptoms were released after absolute diet and anticoagulant treatment 2 days later. There was no postoperative stroke presented in this study.

**Table 2:** Operative and postoperative characteristic per surgical approach.

**Variable**	**TEVAR simultaneous with EVAR** **(*n* = 31)**	**TEVAR separately with EVAR (*n* = 22)**	***P*-value**
Procedure time, minutes	139.5 ± 24.08	172.04 ± 28.04	<0.0001
Contrast, ml	96.45 ± 19.24	92.72 ± 14.20	0.4447
LSCA coverage	3 (9.7%)	2 (9.5%)	-
ICU LOS, days	0.90 ± 2.53	1.36 ± 4.1	0.6158
LOS, days	8.90 ± 2.97	15.81 ± 2.63	<0.0001
Postop respiratory complication	3 (9.7)	1 (4.76)	0.903
CHF	1 (3.23)	1 (4.76)	1.0000
Postop dysrhythmia	3 (9.7)	2 (9.5)	1.0000
Postop MI	2 (6.45)	2 (9.5)	1.0000
Renal failure requiring hemodialysis	2 (6.45)	1 (4.76)	1.0000
Bowel ischemia	1 (3.23)	1 (4.76)	1.0000
Transient SCI	2 (6.45)	1 (4.76)	1.0000
30-day mortality	1 (3.23)	1 (4.76)	1.0000

*LSCA, left subclavian artery; ICU, intensive care unit; LOS, length of stay; CHF, Congestive heart failure; MI, myocardial infarction; SCI, spinal cord ischemia; Data presented as number (%) for categorical variables and as mean ± standard deviation for continuous variables*.

### Primary and Secondary Outcomes

No significant differences were found in the incidence of in–hospital complications between the patients with one-staged repair and two-staged repair. Specifically, each group had one patient who had postoperative SCI, but the symptom of paralysis disappeared and the strength of the lower extremity was recovered after CSF drains. The incidence of postoperative SCI was not significantly different between the two groups (6.45 vs. 4.76%; *p* = 1). The rates of postoperative dysrhythmia, myocardial infarction, congestive heart failure, renal failure, and bowel ischemia were similar between the groups. However, the rate of postoperative LOS was less for the patients with one-staged aortic repair (8.9 ± 2.97 vs. 15.81 ± 2.63, *p* < 0.0001).

#### Follow-Up

**Figure 1:**
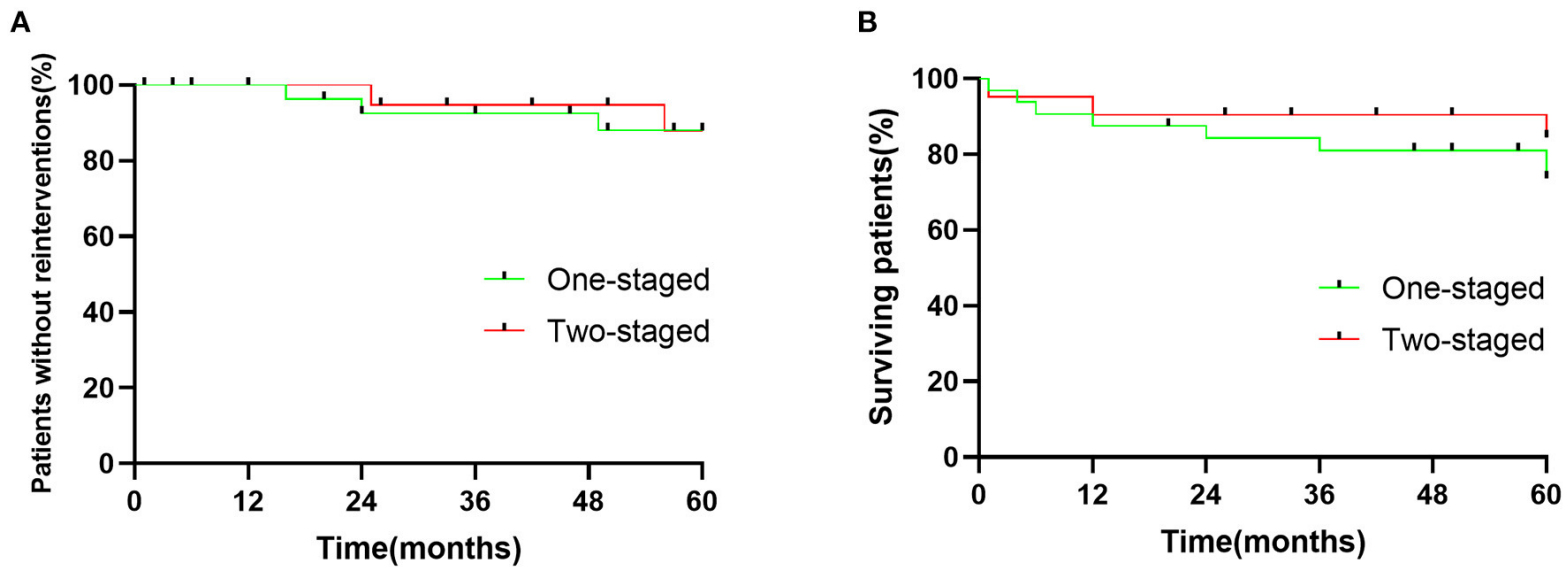
**(A)** Cumulative Kaplan-Meier estimate of patients without reinterventions after endovascular repair of multilevel lesions via one-staged and two-staged repair. *p* = 0.9006. **(B)** Cumulative Kaplan-Meier estimate surviving rate of patients after endovascular repair of multilevel lesions via one-staged and two-staged repair. *p* = 0.4939.

Mean follow-up in one- and two-staged repair groups were 49.25 ± 19.6 and 50.7 ± 17.7 months, respectively. There were no patients with aortic rupture or late aneurysm morbidity in the one-staged group. One death occurred 4 months of postoperative TEVAR repair while waiting for the second repair due to the AAA rupture. Three patients in a one-staged repair group required hemodialysis during follow-up since the progress of the preoperative chronic renal disease. Freedom from re–intervention for one-staged and two-staged groups were 100 vs. 100%, 92.4 vs. 95%, and 88 vs. 88%, at one, two, and 5 years, respectively (Figure 1A). The survival rate for patients who underwent one-staged vs. two-staged aortic repair was 86.4 vs. 90.5%, 87 vs. 90.5%, 76 vs. 84%, at one, two, and 5 years, respectively, with no statistical difference (Figure 1B).

## Discussion

Aortic disease processes are often chronic, but the symptoms may come suddenly which can cause severe outcomes. Endovascular repair is well–known and has gradually become the main method for treating aortic diseases since it has less invasion compared to traditional open surgery (13–15).

Historically, aortic multilevel lesions have been treated by a one-stage procedure with a combination of hybrid open and endovascular approaches; (5–7, 16). however, few cases regarding the total endovascular repair for both aortic segments, with the majority describing two-staged repairs. For two-staged repairs, patients need to undergo two procedures which brings a higher medical expense and more trauma either physically or psychologically. Furthermore, it can expose them to a risk of rupture of the unrepaired aortic lesions while awaiting second–stage treatment. It is reported that 30% of early postoperative deaths after isolated repair of thoracic pathology were caused by the rupture of an untreated infrarenal aneurysm (17).

It is well-known that SCI is a severe complication of TEVAR since the blood supply to the spinal cord was blocked by stent grafts during the procedure. Scali et al. had reported that the patients who had developed SCI (either permanent or transient) have worse survival compared with the non–SCI patients, highlighting the importance of preventing this complication (18).

Previous studies have shown that prior AAA repair is an independent risk factor for SCI after TEVAR (9–21). They believed the infrarenal aortic repair may injure the pelvic collateral blood supply to the distal spinal cord (22–25). Schlösser et al. reported 72 TEVAR patients with prior AAA repair had a significantly increased risk of postoperative SCI compared with patients without prior AAA repair (21). While, other studies have suggested that either repaired or unrepaired AAA was associated with the occurrence of SCI. Accordingly, it seems like the patients would have a higher risk if they have AAA whether they are associated with or without prior AAA repair. In the present study, only left one factor which is the different surgical methods affect the results. For the present study, two patients who underwent simultaneous repair suffered from postoperative SCI which is higher than the two-staged group but had no significant difference. However, the symptom of these two patients was back pain, and they were diagnosed with Stanford type B aortic dissection coexisting with AAA. Multivariate analysis has shown that patients who had undergone elective TEVAR and/or complex EVAR repair were less likely to develop SCI, suggesting that emergent repair is an increased risk factor for SCI. In addition, some imaging studies have reported that aortic pathologies affect the blood supply to the spinal cord altering them to varying degrees, especially in aortic aneurysms associated more often with lumbar and intercostal artery occlusion was caused by atherosclerotic disease (23, 26–29).

Theoretically, the more extensive aortic were covered, the higher the risk of spinal cord ischemia will be. Therefore, simultaneous TEVAR and EVAR may pose a higher risk for the development of paraplegia. In the present study, there is no significance between simultaneous TEVAR and EVAR groups and one-staged group for the SCI occurrence rate. However, the patients who had developed SCI were the patients who were implanted with more aortic stent grafts, which was consistent with the previous studies. Another factor associated with SCI is hypotension, it would be a higher risk for paraplegia if the mean perioperative arterial pressure is <70 mmHg (30). Thus, maintaining the systolic blood pressure of about 140 mmHg and a mean arterial pressure over 75 mmHg was performed during the perioperative period (31).

Some studies reported that preoperative CSF drainage may prevent SCI development. However, there is no sufficient evidence to suggest that preoperative CSF drainage is necessary for endovascular repair. Moreover, preoperative CSF drainage may cause other relative complications. Recent studies have shown that the high incidence of the drain was not obvious functional and the development of spinal hematoma resulting in permanent neurological impairment has prompted a reconsideration of routine use of this adjuvant (32–34).

Previous studies have mentioned that the contrast load will be increased during the simultaneous repair, which is harmful to renal function and potentially causes acute renal failure postoperatively. Additionally, one-staged repairs prolong the operative time in patients who are less conditioned and have multiple comorbidities, which are all associated with long–term progressive renal decline (35). Despite these concerns, in the present study, there was no significant difference in the relative renal function decline (Cr and BUN) between the one-staged and two-staged repair although the Cr and BUN were increased slightly.

The incidence of in–hospital complications was comparable between patients who underwent one-staged and two-staged repair in the overall cohort. However, despite this, the 30-day mortality was increased and the survival rate was decreased in the two-staged group, suggesting that multiple factors contribute to these outcomes. This factor can be attributed hypothetically to the patient-related factors like the patients with one-staged repair are more elderly with less conditioned and have multiple comorbidities. The ICU LOS and postoperative hospital LOS in patients with simultaneously TEVAR and EVAR were significantly shorter than the patients who underwent two-staged TEVAR and EVAR.

The present study has a series of limitations. Firstly, there is a selection bias for the groups of simultaneous repair and staged repair. Secondly, based on the observational nature of the retrospective study, the present study was subject to information and missing data points and reporting bias like the data collection relying on accurate record–keeping from others. Thirdly, the study did not take into account the learning curves of different operators. Moreover, the total number of patients is relatively small in a single center with a short follow-up.

## Conclusion

In conclusion, the results from the present study have shown that simultaneous TEVAR and EVAR can be performed successfully with minimal morbidity and mortality. Although patients with one-staged repair have more risk factors for postoperative SCI, the one-staged repair was not associated with an increased risk of SCI. However, further studies to evaluate additional contributing factors to perioperative mortality in patients with multilevel aortic diseases to improve the short– and long–term outcomes were required.

## Data Availability Statement

The data used to support the findings of this study are available from the authors upon request.

## Ethics Statement

The studies involving human participants were reviewed and approved by Institutional Review Board at the Second Xiangya Hospital of Central South University. The patients/participants provided their written informed consent to participate in this study. Written informed consent was obtained from the individual(s) for the publication of any potentially identifiable images or data included in this article.

## Author Contributions

WZ and CS designed and interpreted the complete data and were major contributors to writing the manuscript. LZ and XL played a leading role in patient enrolment and conducting of the study. JQ, ML, and MW collected and analyzed the patient data regarding clinical characteristics. XL and CS supervised the study and were responsible for funding support. All authors read and approved the final manuscript.

## Funding

This study was supported by the National Natural Science Foundation of China (82120108005).

## Conflict of Interest

The authors declare that the research was conducted in the absence of any commercial or financial relationships that could be construed as a potential conflict of interest.

## Publisher's Note

All claims expressed in this article are solely those of the authors and do not necessarily represent those of their affiliated organizations, or those of the publisher, the editors and the reviewers. Any product that may be evaluated in this article, or claim that may be made by its manufacturer, is not guaranteed or endorsed by the publisher.
